# Case of Alledged Mal-Practice in Surgery

**Published:** 1847-09

**Authors:** 


*Case of alledged Mal-practice in Surgery.*—A resident of Argyle, Washington County, N. Y., in December, 1844, had his limb caught by a falling tree, so as to produce a comminuted fracture of the tibia, and a simple fracture of the fibula, at a point about midway between the knee and ankle joints. How soon the physician who took charge of it, and against whom legal proceedings were commenced for alleged mal-practice, saw it, after the accident, I did not learn; but was informed by the physician himself, that he reduced the fracture, and dressed the limb with compresses, four splints, and bandage. The two lateral extended from the knee to the foot, and the anterior and posterior were somewhat shorter. That which rested upon the contused soft parts, and the comminuted, or detached fragment of the tibia, was removed the next day, in consequence of the existence of a high degree of inflammation, which it seemed to produce, or at least tended to keep up. The posterior and two lateral splints were used during the whole treatment while under the care of the attending physician, which was twelve weeks, as I was informed.

At the end of this period, the limb appeared to be doing so well, that the patient considered the services of the doctor no longer necessary; who, of course, took his leave of the case, though the patient had on the same splints and dressings which had been used in the treatment of the case, from the second day after the receipt of the injury. I was also informed that the patient, pretty soon after discharging the physician, began to use the limb, and that, in planting time, he was hobbling about in the fields, trying to work. From this time, up to about the middle of August, or perhaps later, nothing definite, either with respect to the condition of the limb, or the use to which the patient subjected it, could be learned. At all events, it was clear that the limb was not gaining strength and usefulness as fast as was desirable. or as is usual; whereupon he submitted it to an examination of three or four of the neighboring physicians, who discovered that the fracture was not consolidated in the tibia. In short, preternatural motion was distinct and quite perceptible. I was also informed (but whether true or not, I cannot say, at least I should hope that no such declaration was made), that one of the physicians who examined the case, remarked, in the presence of the patient, that "the attending physician ought to be ashamed of it, and that he ought to pay him for a year's work."

On the 16th of September, 1845, the patient came to Albany, and put himself under my professional care from that date to the 29th of October. At that time the general shape and condition of the limb was as good as the average of cases in comminuted fracture, with the exception of a yielding of the tibia at the lower point of the detached fragment. In short, there was a tardy and fibrous re-union; nothing, however, like an artificial joint.

I treated the case by blisters, iodine, compression, and firm and unyielding splints. While the patient was under my, care, consolidation with bony deposit was evidently, though slowly, taking place, and I had no doubt, when the patient left me, but that, in time, a perfect bony union could be effected. The patient was either unable or unwilling longer to remain under my care.

In the spring of 1846, the doctor was sued for mal-practice; but upon what testimony, or grounds of complaint, the plaintiff or his attorney rested for damages, I have never learned. The cause was to have been tried at the last October circuit court of Washington county; and, on the part of of defence, I was subpœnaed to attend court at that time. It was the week of the commencement of lectures in our College; and as I had just returned from attending court, for nearly a week, at Delaware County, on similar business, I peremptorily refused to obey the summons. The consequence was, that the physician deemed it prudent and expedient to swear the cause off, till the next term, which commenced its session on the 14th ultimo, at which time, on examining the limb of the patient, the bone was found perfectly consolidated, firm and strong, with but little thickening, or bulging, at the seat of the fracture, and not more than a finger's breadth shorter then the other limb.

After some of the medical witnesses had expressed a decided opinion to the plaintiff and his counsel, that he could not obtain a verdict against the physician, on the advice of the counsel the suit was discontinued, by agreement that each party should pay their own costs. The defendant was induced to accept such a proposition, even though he was morally certain, that if the cause had been carried to trial, the verdict would have been in his favor, rather than be obliged to pay the whole costs, as he would have been, because the plaintiff was not in possession of a dollar's worth of property, beyond that exempted by statute,

The doctor was prepared to show by witnesses and good authority, that the tardy union, in this instance, could be attributed to other causes than ignorance or negligence on his part. One of the best authorities, to which reference was had, was a paper written by Dr. George W. Norris, one of the Surgeons of the Pennsylvania Hospital, and published in the American Journal of Medical Sciences, New Series, Vol. III. Dr. Norris, in the introduction to his article, remarks, "Few subjects in surgery possess so much interest and importance, or have more justly exercised the pens of writers, than injuries to the bones, with their consequences; and yet we find, at this day, many points in relation to them demanding farther investigation. Of this kind is that state of the parts following upon a solution of continuity in the bony structure, termed ununited fracture; the causes, pathology, treatment, &c., of which are all matters upon which indefinite ideas are held by the great mass of the practitioners."

Dr. Norris' paper is concise and clear; and yet sufficiently full, upon all important points, for all practical purposes. Tables of cases of fracture, from quite a number of authorities, and an analysis of them, are furnished, to show that non-union of fractured bones does not often occur; and yet oftener than one would infer, if he were to judge merely from a limited personal observation, or from the reported cases. In relation to this point, Dr. Norris remarks, "In surgery, unhappily, we are all too prone to silence in regard to our unfortunate cases, while it is rare that success after an operation at all out of the common course, is not made known." According to references introduced by Dr. Norris, one authority asserts, that non-union of fractures does not occur oftener than six or eight times in a thousand; and another says that it happens only five or six times in nearly four thousand cases.

I do not pretend that my field and opportunities for observation are anything like those of surgeons who have, for many years, been connected with large hospitals; and yet I think I can recall to mind some ten or twelve cases of fracture, where either a tardy union, or a fibro-ligamentous union, or a false joint, was the result. The first, fracture and non-union of the bones of the fore-arm, came under my observation about thirty years ago, while I was a pupil. The second was a perfectly-formed artificial joint, which existed in the humerus of a man, who had been a soldier in the army of our last war with Great Britain. In the third case, there was fracture of the bones of both legs below the knee. In one, bony union took place readily; but in the other, the tibia remained moveable for many months, and whether it ever became firm and consolidated, I never learned. The fourth case fell under my observation while I was connected with a medical school in Vermont. It was a fracture of the femur, just above the patella, which had been, for many months, treated for a dislocation. An attempt was made to effect bony union by pressure and splints; but whether successful or not, I cannot say. The fifth case happened to a laboring man, "a clay digger," who was caught under a mass of earth, and received a severe contusion of his shoulder, and fracture of the ulna near its middle. The pain and swelling seemed to be altogether confined to the humerus and shoulder-joint. In that situation no discoloration, or fracture, could be detected. In a short time the patient again resumed the use of his spade. After he had been engaged in this way for some time, he complained of weakness of the fore-arm, and on examination, a fracture of the ulna, and non-union, were discovered. Whether any treatment was adopted to effect union, and with what result, I know not. The sixth case was one in which there was a fracture of the radius and ulna in two places—one united, and the other did not. The seventh case might be called one of tardy union of the thigh. At the end of twelve or fourteen weeks, the bone yielded in the seat of the fracture very freely. A starch bandage was applied, and I was informed that a cure was effected. Something over a year since I saw another case of what would be called tardy union of fracture of the tibia. At the end of three or four months no bony callus had formed. In about a year the fracture became firm. In the ninth case there was perfect pseudarthrosis of the femur, within two inches of knee-joint, which had existed two or three years. Without the aid of something to stiffen the limb, the patient was unable to walk.

The tenth case occurred in my own practice, about a year and a half ago. The subject of it, in a drunken frolic, was thrown into deep snow, with one or two persons upon him, by which an oblique fracture of the tibia and fibula was produced. A sharp point of the tibia perforated the skin. The small wound, thus produced, healed by the first intention; though some ten days after, a moderate degree of suppuration took place over the extreme point of the bone; not, however, at the point where the skin was punctured. At the end of two months no bony callus had formed, though the patient had been kept quiet in bed. Blisters, iodine, compression, and firm dressings, constituted the subsequent mode of treatment for five or six months. At this time the patient again returned to his old habit of intoxication; and, of course, took himself out of my hands, with a fibro-ligamentous union of the tibia. I have not seen the patient for the last two or three months, so that I cannot now speak of the present condition of the limb.*

The eleventh case is the subject of this report, whose history and result have already been given.

---

*Since the above was written, I met with the patient upon the side walk, with one crutch, which seemed to be quite as necessary to preserve his equilibrium, as respected his body, as to supply the place of a defective limb. However, to show me that he could progress with his organ of locomotion, he lifted it, and hobbled off tolerably well.

The twelfth and last case that I can now bring to mind, is a fracture oi the femur, within an inch and a half of the patella, which occurred in the first week in May last, in my absence in attendance at the late National Medical Convention. It has been in charge of one our best physicians and surgeons; and I have aided twice or three times in dressing it. The straight position, permanent extension, long splint, compresses and roller, constituted the plan of treatment; and yet, up to this date, no bony union has taken place.

It will be perceived that in the fourth, ninth and twelfth cases, there is a great similarity—all fractures of the lower part of the femur; and, in all probability, extending into the bursa, just above the patella, or into the cavity of the joint, which may have been one of the chief causes of non-union.

I might have mentioned, in connection with an account of the elbow mal-practice suit (the subject of my former communication), a curious coincidence. On returning from Delaware county, last September, in company with Mr. Tabor, the counsellor for the plaintiff, with mutual regrets that the suit could not have been tried, and on my part with almost a determination not to go again, a lad, about twelve years of age, came to my office within half an hour after my arrival, with what I considered to be a case of injury of the elbow-joint, of precisely the same kind as that of the "possee man." The boy was sliding down the banister of a long flight of stairs in our Exchange building, and, in some way, his arm passed between the rounds, while the weight of his body was brought to bear upon the joint, so as to produce a fracture of the external condyle of the humerus, and a posterior dislocation of the ulna. The nature of the injury was so susceptible of being demonstrated, and so interesting, as it related to the case under legal investigation—and with a view to furnish testimony which would do away with the necessity of my making another tedious journey to attend court again in Delaware county, I immediately sent for Mr. Tabor, before reducing and dressing the dislocation and fracture. After pointing out the several points of distortion to Mr. T., and the students in my office, I preceeded to reduce it, by grasping the fore-arm with one hand, and the lower part of the humerus with the other. *Forcible flexion*, conjoined with extension and counter-extension, readily brought the displaced parts into their proper position. A compress was applied to the fractured condyle and secured by a roller, and the limb kept, by a sling, in a position somewhat more than at a right angle. It has resulted in a very good cure. Flexion is perfect, and extension nearly so.

I know of no text-book in surgery, which mentions injuries of the description of these two cases; and yet the general principle is inculcated in all standard surgical works, of reducing the dislocation first, when dislocation and fracture co-exist.

Legal prosecutions for mal-practice in surgery occur so often, that even a respectable surgeon may well fear for the results of his surgical practice. We must confess, however, that too many ignorant and careless men get into the ranks of our profession, who are liable to commit errors, for the consequences of which, the law holds them responsible. This would seem to indicate the necessity of higher attainments in our profession.

In several instances I have known suits to be sustained against physicians and surgeons, for not reducing a dislocated joint; but very seldom for the mal-treatment of fractures. In the treatment of fractures more or less change from the normal condition is a natural consequence, whether treated in the most approved and successful mode or not. Nor such doubt or uncertainty need be the consequence of a dislocation. If a dislocation be overlooked, or not reduced, the charge of negligence or ignorance is much more likely to be sustained. As the people, or, at least, that class of persons who are most exposed to accidents, and the least responsible, either for the surgeon's bill for professional attendance, or for the costs of a suit for mal-practice, seem to require high surgical attainments, would it not be well, in obscure and doubtful cases, to divide the responsibility, by having the advice and assistance of the best surgeon that could be obtained. At all events, I would advise, that a physician or two, or one or two medical students, be invited to be present at the examination of the case; to hear the nature of it explained, and the probable prognosis; and to observe the course of treatment, as much as practicable. The profession must *respect* and *protect* themselves, exercise a spirit of liberality towards each other, and, at the same time, not deprive the most humble and unfortunate sufferer of his rightful claims for justice at their hands. ALDEN MARCH

*Albany, N. Y., July*, 1847. [*Boston M. and S. Journal.*

